# The intensity of coronavirus anxiety and its associations with depressive symptoms and burnout among Polish nurses and firefighters: a preliminary cross-sectional study

**DOI:** 10.1192/j.eurpsy.2022.1295

**Published:** 2022-09-01

**Authors:** H. Sienkiewicz-Jarosz, Ł. Mokros, J. Januszczak, Ł. Baka, P. Świtaj

**Affiliations:** 1 Institute of Psychiatry and Neurology, 1st Department Of Neurology, Warsaw, Poland; 2 Medical University of Lodz, Department Of Clinical Pharmacology, Łodz, Poland; 3 Institute of Psychiatry and Neurology, 1st Department Of Psychiatry, Warsaw, Poland; 4 Central Institute for Labour Protection – National Research Institute, Department Of Social Psychology, Warsaw, Poland

**Keywords:** Anxiety, burnout, Coronavirus, depressive symptoms

## Abstract

**Introduction:**

Nurses and firefighters are professions characterized by exposure to high occupational stress.

**Objectives:**

The purpose of the present study was to investigate the intensity of coronavirus anxiety and its associations with depressive symptoms and burnout in samples of Polish nurses and firefighters during the COVID-19 pandemic.

**Methods:**

Fifty nurses and 55 firefighters were recruited for the study. Respondents were administered the Coronavirus Anxiety Scale (CAS), the Center for Epidemiologic Studies Depression Scale-Revised (CESD-R) and the Oldenburg Burnout Inventory (OLBI). Descriptive statistics, Pearson product-moment correlations and one-way ANOVA were used to analyze the data.

**Results:**

Nurses scored significantly higher than firefighters on the CAS (*M* = 2.76, *SD* = 4.18 vs. *M* = 1.15, *SD* = 2.24; *p* < 0.05), the CESD-R (*M* = 11.64, *SD* = 10.80 vs. *M* = 5.85, *SD* = 7.34; *p* < 0.01) and both dimensions of the OLBI, i.e. exhaustion (*M* = 2.38, *SD* = 0.56 vs. *M* = 1.91, *SD* = 0.55; *p* < 0.001) and disengagement from work (*M* = 2.28, *SD* = 0.48 vs. *M* = 1.93, *SD* = 0.43; *p* < 0.001). In the both study groups coronavirus anxiety significantly correlated with depressive symptoms and exhaustion, and only in nurses also with disengagement from work.

**Conclusions:**

This study demonstrated that coronavirus anxiety was more pronounced in nurses than in firefighters. The findings provide preliminary evidence for the positive associations of coronavirus anxiety with depressive symptoms and burnout in both groups.

**Disclosure:**

The study was supported by the grant of the National Centre for Research and Development (NCBR) No. I.PB.08 “Occupational burnout and depression in professions with exposure to high levels of occupational stress: determinants, prevalence, interrelations a

